# Phytoliths in Taxonomy of Phylogenetic Domains of Plants

**DOI:** 10.1155/2014/648326

**Published:** 2014-08-27

**Authors:** Kirill S. Golokhvast, Ivan V. Seryodkin, Vladimir V. Chaika, Alexander M. Zakharenko, Igor E. Pamirsky

**Affiliations:** ^1^Scientific Educational Center of Nanotechnology, Far Eastern Federal University, 10 Pushkinskaya Street, Vladivostok 690990, Russia; ^2^Laboratory of Ecology and Protection Animals, Pacific Institute of Geography FEB RAS, 7 Radio Street, Vladivostok 690041, Russia; ^3^Laboratory of Enzyme Chemistry, Pacific Institute of Bioorganic Chemistry FEB RAS, 159 Prospect 100 Let Vladivostoku, Vladivostok 690022, Russia; ^4^Laboratory of Molecular Biology, Blagoveshchensk State Pedagogical University, 104 Lenina Street, Blagoveshchensk 675000, Russia

## Abstract

We discuss, from the aspect of phylogeny, the interrelationships of the phytolith types in plants from the main taxonomical groups (algae, lichens, horsetails, gymnosperms, and floral plants) with homologues of known proteins of biomineralization. Phytolith morphotypes in various phylogenetic plant domains have different shapes. We found that, in ancient types of plants (algae, horsetails, and gymnosperms), there are fewer different phytolith morphotypes compared to more modern plants (floral plants). The phytolith morphotypes in primitive plants are generally larger than the morphotypes in more highly organized plants. We found that the irregular ruminate and irregular smooth morphotypes are the two most frequently encountered phytolith morphotypes in the tested plants (from algae to floral plants). These two morphotypes probably have a universal role. Silacidins, silicon transporters, silicateins, silaffins, and silicase homologues are often found in the major taxonomic groups of plants. Red algae had the smallest number of homologues of the biomineralization proteins (70–80), Monocotyledonous: 142, Coniferous: 166, Mosses: 227, and Dicotyledones: 336.

## 1. Introduction

Silicon is an extremely important element in all plants [1–5]. Phytoliths, which are specific biomineral silicon formations, are found in many groups of plants [6–8], and the roles and functions of phytoliths are varied [9, 10]. They provide defense from predators [11, 12], carry out optical functions in plants [13], and provide structural and frame elements, which contribute to stalk and leaf stiffening [10].

The role of phytoliths in the life of plants is extremely important, and the biomineralization process and phylogenetic history are evolutionarily fixed in plants.

Red algae and lichens are among the most ancient forms of plants [14–20].

Red algae appeared approximately 1 billion years ago [18, 20–22].

Lichens, according to paleontological findings, emerged in the early Devonian Period (approximately 400 million years ago) [23] or slightly earlier [24–27]. The first lichens might have been aqueous [28].

Horsetails arose in the top Devonian Period and developed from currently extinct rhyniophytes (Rhyniales) [18, 29, 30].

Gymnospermous plants appeared at the end of the Devonian Period, approximately 370 million years ago [29, 30].

Brown algae comprises a relatively young group of organisms and date approximately from 150 million years ago [31] to 200 to million years ago [32]. Evolutionarily, brown algae comprise a unique group of live organisms because they developed from eukaryotes and have a multicellular body structure; they have survived to the present.

For studying the development and regularities of biomineral processes in a phylogeny of plants, it is necessary to study the most ancient forms (algae and lichens) and then track their metamorphoses into modern (floral) plants.

This work aims to analyze the morphogenesis of plant phytoliths from the aspect of phylogenetics.

## 2. Materials and Methods

### 2.1. Phytolith Analysis

The samples of land plants were collected near the Sikhote-Alin Biosphere Reserve (Primorsky region, Russia), and the mature algae originated in the Sea of Japan (Peter the Great Bay). The samples of phytoliths were prepared within 2 days after the collection of the plants. The phytoliths were collected according to the technique of Piperno [7]. The requisite part of the plant was heated for 4 hours and then washed with a 10% HCl solution and was followed by washing with concentrated nitric acid and distilled water. The sediment was centrifuged by an OPN-8 centrifuge (“Dastan,” Bishkek, Kyrgyzstan) for 10 minutes at 1000 g and then selected for microscopic research using an AxioScope A1 light microscope with an AxioCam 3 digital video camera (Zeiss, Oberkochen, Germany). The definition of the phytolith morphotypes was carried out using the International Code for Phytolith Nomenclature 1.0. [8]. For the analysis, 150 phytoliths of each plant were selected. The statistical analysis was performed using Biostat software with an assessment of the statistical importance of the indicators.

### 2.2. In Silico Analysis

The search for homologues of typical representatives of silicon transporters (SITs), aquaporins, silaffins, silicateins, silacidins, and silicase (peptides and proteins of diatoms, sponges, rice, and corn were chosen), in the nucleotide sequence bases, was conducted using BLAST (http://blast.ncbi.nlm.nih.gov) as described in the paper [33]. From the Uniprot base (March 2014), we obtained the amino acid sequences of SIT (ID O81199 and C7G3B4), aquaporin (ID Q6Z2T3), silaffins (ID Q9SE35, Q5Y2C0 and Q5Y2C2), and silicateins (ID B5B2Z1, B1GSK9, and B5LT52). The sequences of silacidins were obtained from the work [34], and those from silicase were taken from [35].

## 3. Results

We hypothesized that there might be communication between known biosilicification proteins and/or their homologues and phytolith morphotypes in plants of different phylogenetic origin.

There are no studies regarding representative silaffins, silacidins, silicateins, and silicases (except research concerning aquaporins) in plants. We attempted a computer search (in proteome and genome databases) of the genes and homologues of the proteins identified above (Table 1).

**Figure 1:**
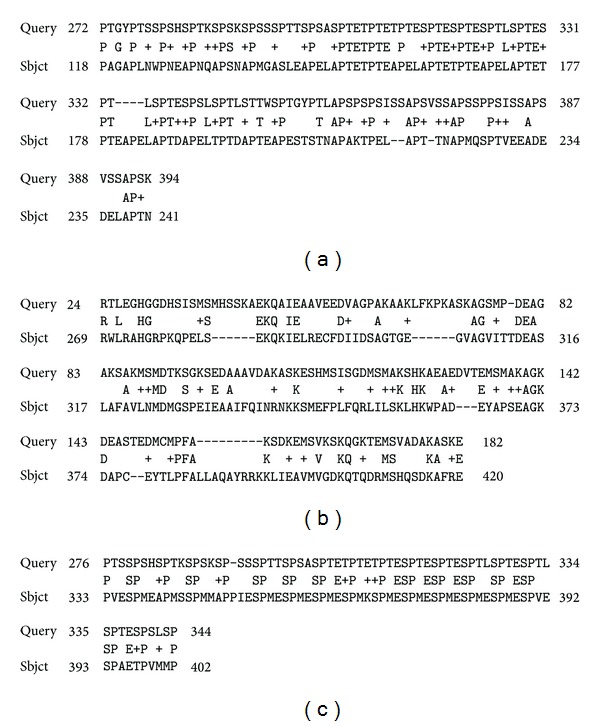
Examples of the alignment of the polypeptide chains of silaffins and the proteins of plants. (a) silaffin of diatom* T. pseudonana* (ID Q5Y2C2, Query) and an unknown protein of a soy bean* G. Max* (Sbjct); (b) silaffin of diatom* T. pseudonana* (ID Q5Y2C0, Query) and an unknown protein of the moss* P. Patens* (Sbjct); (c) silaffin of diatom* T. pseudonana* (ID Q5Y2C2, Query) and an unknown protein the moss* P. patens* (Sbjct).

**Figure 2:**
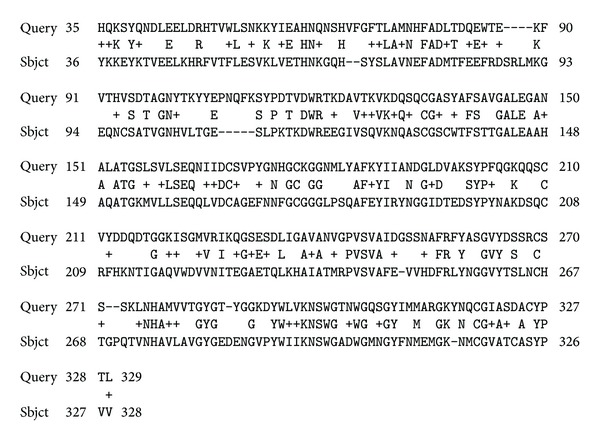
Example of the alignment of the polypeptide chains of silicatein of a sponge,* L. oparinae *(ID B5LT52, Query), and an unknown protein of the moss* P. patens* (Sbjct).

**Figure 3:**
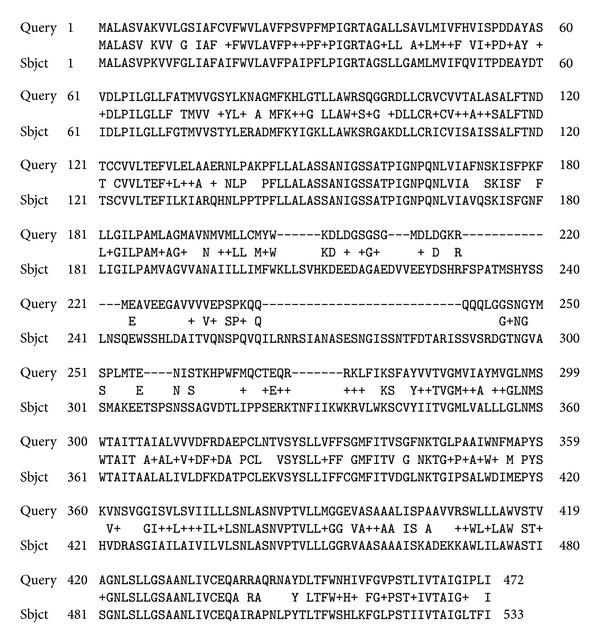
Example of the alignment of the polypeptide chains of the SIT of corn,* Z. mays* (ID C7G3B4, Query), and an unknown protein of soy bean* G. Max* (Sbjct).

The genomes of all of the studied species of plants contained genes of aquaporins (SIT) and/or their homologues (a high degree of identification of primary structures). Some of these aquaporins have been identified previously (database information). No silaffins, silacidins, silicateins, or silicases were found. However, many short and long fragments of various proteins (some almost whole proteins) (Figures 1, 2, and 3) were homologous to these proteins.

It should be noted that, for all of the studied plants, homologues with existence conserved based pairs of length 3–6 amino acids were observed most often, and longer stretches were rarely observed.

The most characteristic homologues for silicateins were various cathepsins, and carbohydrases were the most characteristic homologues for silicase. Structural homologues of the silacidins presented with very short parts of the amino acid chains of various proteins: unknown proteins, histone deacetylase proteins, xylosidase, photosystem proteins, synthetase, gigantean, DNA binding proteins, oxidase, transcription factors, RNA polymerase, floral homeotic proteins, synthase, and gamma-gliadin.

Only one protein from among the homologues of silacidins participates in a biomineralization: dentin sialophosphoprotein-like of soy bean. The silaffin homologues are represented by unknown proteins, ATPases, transcription factors, kinases, dehydrogenases, synthases, seed maturation proteins, and glucosidases.

The low degree of reliability or no reliability, which is reflected in high E values, is caused by the small length of the homologous chains in most cases. Low level or no reliability was characteristic of silacidins, in which the length of the homologous parts does not exceed 2–20 base pairs. The obtained data are of interest, in particular to matrix peptides and proteins of a biomineralization, silaffins (Figure 1) and silacidins.

The existence of a certain zwitterionic structure [36, 37] in a polypeptide chain (by analogy with silaffins and silacidins) of most of the identified proteins indirectly points to the possibility of their participation in the biosilicification process as catalysts of the sedimentation of biosilicon dioxide. This finding requires further study.

The results of the percentage of the phytolith morphotypes of all of the studied plants are given in Table 2.

**Figure 4:**
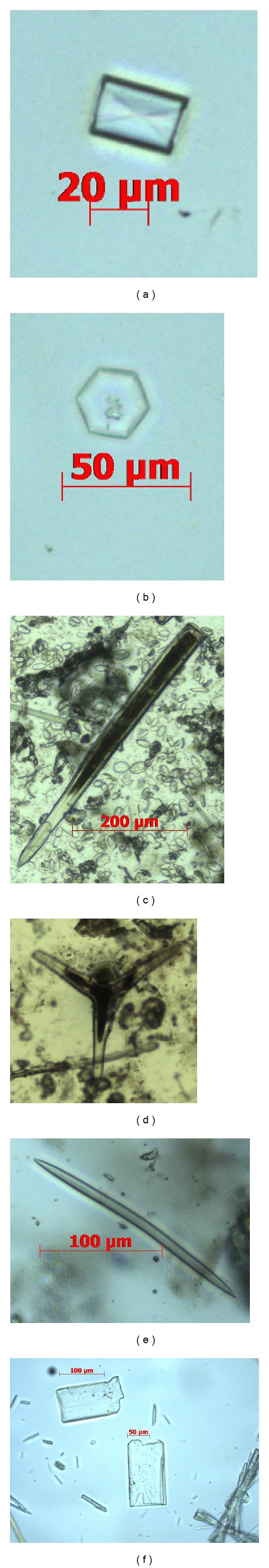
The most characteristic forms of the phytoliths of algae: (a-b)* Tichocarpus crinitus:* (a) pyramid; (b) hexagon, (c)* Laurencio tropica*: (c) hollow needle, (d-e)* Amphiroa anceps*: (d) triple needle; (e) needle, (f)* Fucus evanescens*: (f) long smooth rectangular.

The most interesting specific types of phytoliths of algae are presented in Figure 4.

**Figure 5:**
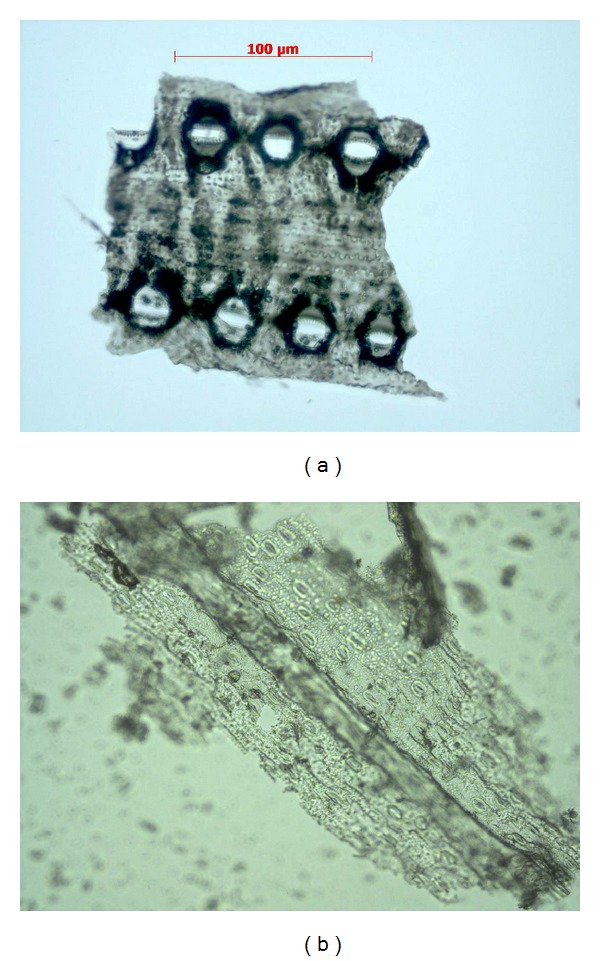
Parts of the silicon armor of* Equisetum hyemale* (a) and* Equisetum fluviatile* (b).

The specific phytolithic profile of the horsetail plant, which is one of the most ancient plants to have survived, is shown in the almost full silicification of the plant surface (Figures 5(a) and 5(b)), which apparently serves as protection against drying and is analogous to the wax layer on floral plants.

**Figure 6:**
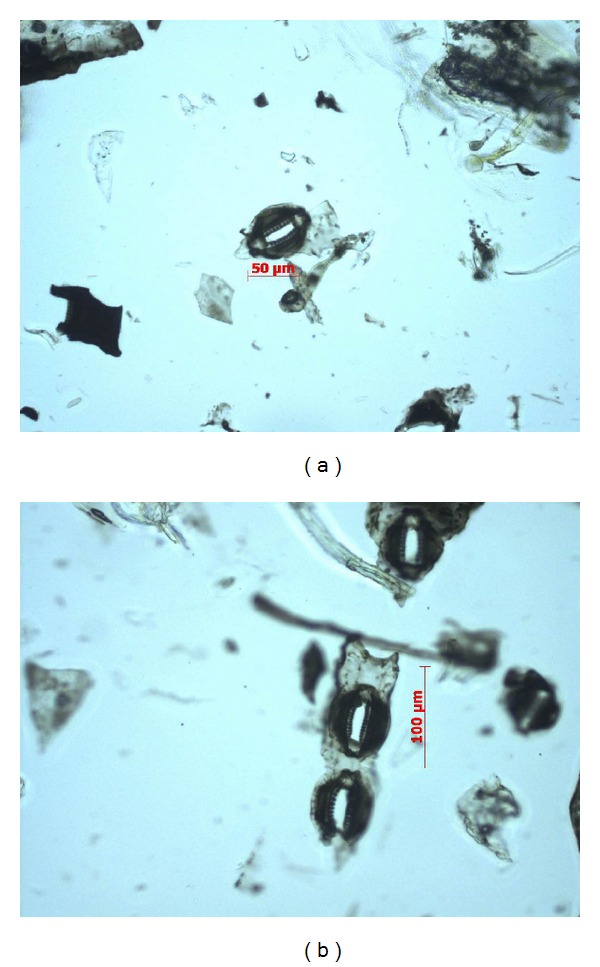
Separately located stomates from the silicon armor of* Equisetum hyemale.*

The phytolithic profile of a horsetail plant predominantly consists of a polylobate, tracheid, and separately located siliceous stomates (Figures 6(a) and 6(b)).

Another specific phytolith of the horsetail plant is the polylobate, which is a structural component of the silicon “armor” that is fastened with “teeth-” like components [38].

**Figure 7:**
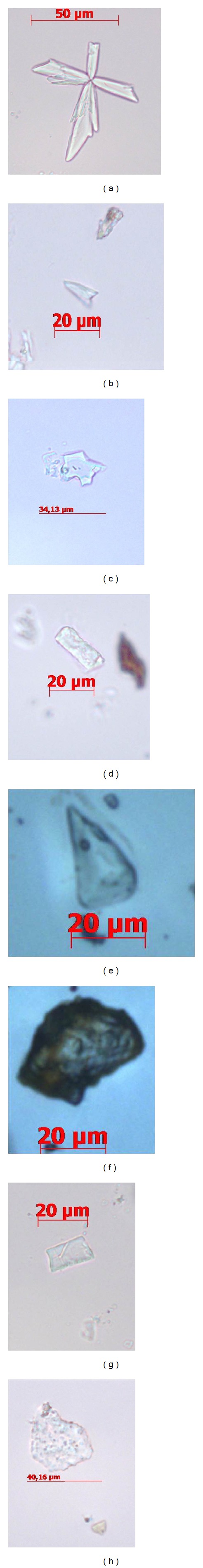
The most characteristic phytolith forms of floral plants were as follows. (a)* Berberis amurensis*: (a) stellatus (meet 4-, 5-, 6-, 7-, and 8-beam differences); (b–d)* Schisandra chinensis*: (b) bark, cuneiform; (c) bark, polyhedron smooth; and (d) leaves, parallelepiped extended rough; (e-f)* Panax ginseng*: (e) leaves, cuneiform; (f) root, irregular ruminate; (g)* Bergenia pacifica*: (g) leaves, parallelepiped smooth; and (h)* Eleutherococcus senticosus*: (h) leaves, irregular ruminate.

In floral plants, we observed a wide polymorphism and ratio of morphotypes (Figures 7(a)–7(h)).

Apparently, more specific types of phytoliths are observed in floral plants than in algae, lichens, and gymnosperms.

## 4. Discussion

**Figure 8:**
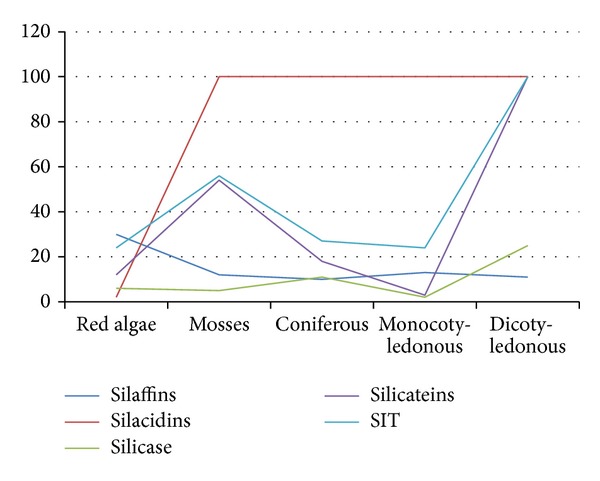
Chart of the quantity of the homologues of the biomineralization proteins in different taxonomical groups of plants.

The results of the comparison of the total number of homologues allowed us to draw some conclusions.  Figure 8 shows the plants in which the homologues of silacidins are observed most often. This group is extremely widespread across the phylogenetic tree of plants. We hypothesized that matrix peptides and proteins (silaffins and silacidins) could be among the most ancient matrix substances of biomineralization [33].

Based on the degree of frequency of the occurrence of homologues, SIT is first, followed by silicateins, silaffins, and silicase.  Figure 8 shows that lines of siliffins and silicateins are similar and demonstrate their joint participation in plant processes.

In the analysis, primitive red algae had the smallest number (70–80) of homologues of the biomineralization proteins of the 4 types. The number of phytoliths morphotypes in red and brown algae (4–6) was insignificant.

In Monocotyledonous, 142 homologues were found. Coniferous contained 166 homologues and from 3 to 5 morphotypes, including a specific type in the shape of a circle. More homologues of biomineralization were observed more in the Mosses than in the other groups (more than 227). Mosses are an ancient deadlocked branch of the highest plants, which appeared at the beginning from algae of the Cambrian Period (approximately 600 million years ago) [39].

More than 336 homologues and from 8 to 12 phytolith morphotypes were observed in Dicotyledones.

Across all of the plants (from primitive algae to floral) a variety of forms (12 morphotypes in certain case) and a large quantity of biomineralization protein homologues were observed.

The morphometric analysis provided additional data indicating that the sizes of the phytoliths of floral plants differ from each other by no more than 10-fold. In primitive plants, particularly in algae and lichens, the difference in the sizes of the phytoliths of similar morphotypes is 2 or 3 and even 4-fold.

The phytoliths in these primitive plants considerably exceed the size of the phytoliths in more complexly organized plants. In* Laurencio tropica* red algae, the surface area of the needle phytolith morphotype is 19489,32 ± 875,75 mkm^2^, which is 10 times larger than the average area of the phytoliths of similar morphotypes in floral plants, and, in particular, in* Eleutherococcus senticosus* (1673,64 ± 1108,22 mkm^2^). The identical difference in the indicators in different plants is observed for other morphometric parameters, including the perimeter, length, and width.

Identical phytolith morphotypes in plants in different ecological niches carry out various functions.

In most cases, in all of the studied plants (from algae to floral plants), there are two main types of phytoliths, irregular smooth (shapeless) and irregular ruminate. These two types have a likely universal role. The structural mechanisms must be universal for all of the groups, as in the case of the implementation of stability and stalk strength. In the structural phytolith group, it is worth considering the extended morphotypes. The synthesis mechanism of the shapeless phytoliths is similar across the entire phylogenetic tree and is possibly connected with silacidins, as their homologues occur most often. Silacidins are small peptides that catalyze the formation and regulate the size of the nanospheres of silicon dioxide from silicon acid and play a central role in the formation of the cellular wall of diatom algae [34].

It is logical to assume that these phytoliths have a general ancient function in all types of plants, which is confirmed by our data. In all primitive plants, shapeless and extended phytoliths (Table 3) prevail.

Specific types of phytoliths unambiguously show specific and probably unique functions in groups of plants that appeared later with evolutionary selection. Klančnik et al. found that the circle- and needle-shaped phytoliths found in some algae could carry out optical functions and focus and redirect sunlight [13].

We demonstrated previously [40] that micrometer silicate aluminosilicate objects could concentrate charges comparable to the membrane potential on the surface (according to our own data, the charge of the particles of quartz of 1–10 microns in size reaches more than −27 mV and that of volcanic glass reaches more than −36 mV). Some phytolith morphotypes in plants could be catalysts of various chemical reactions in the intercellular environment and of supramolecular interactions in the course of ontogenesis.

## Figures and Tables

**Table 1:** The presence of functional and structural homologues of proteins and biosilicification peptides in plants of various types of taxonomy.

	Parameters	Silaffins	Silacidins	Silicase	Silicateins	SIT
*Chondrus crispus* ^ 1^	∗	30 F	2 F	6 F and P	12 F and P	24 F and P
	*E* value	0.046–9.9	2.4–6.3	7*e* ^−04^–7.2	0.78–6.5	2*e* ^−11^–9.8
	%	26–60	35–48	24–48	26–42	23–79

*Physcomitrella patens* ^ 2^	∗	12 F and P	>100 F	5 F and P	54 F and P	56 F and P
	*E* value	1*e* ^−09^–2.4	0.002–3.4	4*e* ^−13^–8.3	1*e* ^−65^–5.6	1*e* ^−80^–9.3
	%	37	32–100	26–35	23–45	23–58

*Pinus pinaster* ^ 3^	∗	10 F and P	>100 F	11 F	18 F and P	27 F and P
	*E* value	0.24–8.3	0.71–1116	0.6–9.2	5*e* ^−28^–10	0.51–69
	%	24–50	26–100	26–40	20–37	19–75

*Triticum turgidum* ^ 4^	∗	13 F	>100 F	2 F	3 F	24 F and P
	*E* value	0.92–9.5	0.002–390	5.1–6.5	0.078–4.2	1*e* ^−20^–9.8
	%	26–31	32–100	31	23–45	18–56

*Glycine max* ^ 5^	∗	11 F	>100 F	25 F and P	>100 F and P	>100 F
	*E* value	1*e* ^−12^–5	0.002–1.80	4*e* ^−16^–9.2	1*e* ^−70^–9	2*e* ^−9^–8.7
	%	24–40	32–100	19–37	22–52	23–67

∗Number of homologues, fragment (F), protein (P), ^1^red algae, ^2^mosses, ^3^coniferous, ^4^monocotyledonous, and ^5^dicotyledonous.

**Table 2:** Phytolith morphotypes from the plants from different taxons.

	Number of morphotypes	Prevailing morphotypes, %	Specific morphotypes, %	Figure
Red algae				
*Tichocarpus crinitus *	6	Irregular ruminate (*41.3*)	Pyramid (*36*) Hexagon (*11.3*)	Figure 4(a) Figure 4(b)
*Mastocarpus stellatus *	4	Irregular smooth (*38*)	Cylinder smooth (*8.7*)	
*Laurencio tropica *	5	Irregular ruminate (*13.3*)	Hollow needle (*8.7*)	Figure 4(c)
*Amphiroa anceps *	5	Irregular ruminate (*46.7*)	Needle (*8.7*) Triple needle (1%)	Figure 4(d) Figure 4(e)

Brown algae				
*Fucus evanescens *	4	Irregular ruminate (*33.5*) Irregular smooth (*25.5*)	Elongate smooth (*5.3*) Elongate rectangle smooth (*4.7*)	Figure 4(f)
*Saccharina latissima *	6	Irregular smooth (25.7) Irregular ruminate (23.7)	Elongate (*19.7*)	

Lichens				
*Cladonia* spp.	4	Irregular ruminate (*56*) Irregular smooth (*28*)	Oval (*4.7*)	
Usnea spp.	5	Irregular smooth (*43.3*) Irregular ruminate (*24.6*)	Circle (*16*)	

Horsetails				
*Equisetum fluviatile *	3	Silicification of a surface of a plant	Polylobate Tracheid Stomata	Figures 5(a) and 5(b) Figures 6(a) and 6(b)
*Equisetum hyemale *	3	Silicification of a surface of a plant	Polylobate Tracheid Stomata	Figures 5(a) and 5(b) Figures 6(a) and 6(b)

Coniferous				
*Pinus koraiensis *	4	Circle (*50.67*) Irregular ruminate (*30*)	Circle (*50.7*)	
*Abies squamata *	3	Irregular ruminate (*68*) Irregular smooth (*28.7*)	Circle (*3*)	
*Juniperus sibirica *	3	Irregular ruminate (*59.33*)	Circle (*2.7*)	
*Larix cajanderi *	5	Irregular ruminate (*62.3*)	Circle (*24.7*)	

Floral				
*Berberis amurensis *	8	Stellatus (*48*) Irregular ruminate (*17*)	Stellatus (*48*)	Figure 7(a)
*Schisandra chinensis *	12	Irregular ruminate (*43*)	Cylinder smooth curved (*10*) Epidermal short cell (*7*)	
*Panax ginseng *	7	Irregular smooth (*35.3*) Cuneiform (*10.7*)	Stomata	
*Bergenia pacifica *	8	Irregular ruminate (*37*) Globular (*20*)	Oblong (*13*) parallel epipedal with the rounded-off edges (9)	
*Eleutherococcus senticosus *	5	Irregular ruminate (*85*)		

**Table 3:** Evolutionary aspects of the morphotypes of phytoliths.

Age	Morphotypes	Plant taxons
Ancient	Irregular smooth, irregular ruminate, needles, elongated, elongate, and geometrically correct (pyramids, hexagons, etc.)	Red algae, lichens
Median	Circular and polylobate, silicification of a surface of a plant	Horsetails, Coniferous plants
Modern	Rectangular, elongated, and polygonal	Brown algae, floral
